# Dental Development in Patients with Hypophosphatemia Rickets

**DOI:** 10.5005/jp-journals-10005-1046

**Published:** 2010-04-15

**Authors:** AI-Jundi SH, Hazza’a AM

**Affiliations:** 1 Associate Professor in Pediatric Dentistry, Department of Preventive Dentistry, Jordan University of Science and Technology Irbid, Jordan; 2 Associate Professor of Oral Radiology, Vice Dean of Dentistry, Department of Oral Medicine and Surgery, Jordan University of Science and Technology, Irbid, Jordan

**Keywords:** Hypophosphatemia, rickets, dental development.

## Abstract

**Background:**

Hypophosphatemic Rickets (HR) is a disease that affects mineralized structures including bone and dentine, studies on dental development in these patients are scarce with equivocal results.

**Aim:**

To assess dental development of a group of children with (HR) and to compare that to healthy matched controls, and to assess relationship between delayed medical treatment and dental development.

**Materials and methods:**

This is a controlled cross-sectional study carried out on a sample of 21 children with HR and healthy age and sex matched controls, diseased children were diagnosed at different ages. Dental age was assessed using Demirjian et al method. The difference between ages of study and control groups was assessed using t-test, Pearson correlation was used to test relationship between age of commencement of treatment and dental development delay.

**Results:**

Most HR subjects demonstrated dental delay ranging from 0.2 to 2.5 years which was significant (p-value = 0.028). The difference between dental age of the study and control groups was statistically significant using paired t-test. There was no correlation between age of commencement of treatment and amount of dental delay.

**Conclusion:**

Dental development was significantly delayed in a group of HR patients compared to matched healthy controls. Delay in commencement of treatment may lead to a permanent deficit in dental development.

## INTRODUCTION

Hypophosphatemic rickets (HR) is of special interest to the dentist due to the dental manifestations associated with this disease. The present report represents part of a study on a relatively large population of children with HR investigating aspects of this disease that were not sufficiently reported in the literature.

The main defect in HR is a phosphorus-wasting disturbance that affects bone metabolism, causing rickets in pediatric patients and osteomalacia in adults.^1^

The genetic mode of inheritance of HR suggest three forms of this disease, with X-linked hypophosphatemic form (XLHR) being the most common at a prevalence of about 1/20,000 live births.^2^

Many dental problems associated with HR were reported including; dental abscesses,^3-5^ enamel hypoplasia,^6-9^ and ectopically erupted permanent canines.^10^ A couple of recent reports by the same author indicated that these patients have a deficiency in the anterior cranial base, class III skeletal relationship,^11^ and reduction in maxillary arch dimensions (unpublished data).

It is also well-established that HR causes mineralization defects in teeth, leading to poorly mineralized and hypoplastic dentin that consists of calcopherites rather than properly mineralized dentin.^8^ Theoretically, this mineralization defect together with hypophosphatemia may affect dental development and maturity in these patients. Only one controlled study is available in the literature on the effect of HR on dental development which found no significant difference in dental age compared to chronological age in both diseased and controls in a sample of 19 patients, the authors suggested that their findings may indicate that medical therapy, which was started early (before age 2 years) in their subjects, may have prevented the derangements of dental development.^10^ Some controversy exists as to weather this disease has an effect on dental development which is corrected by early treatment, or that the disease does not actually affect dental development.

The aim of this study is to assess dental age of patients with HR, diagnosed at different ages, and to compare this to matched healthy controls using Demirjian et al method,^12^ it also aims at establishing the effect of medical treatment on dental development.

## MATERIALS AND METHODS

An institutional review board (IRB) approval was obtained from the ethical committee of Faculty of Medicine in Jordan University of Science and Technology on the study ptotocol. Informed written consent was obtained from parents of all participants. Infection control and radiographic protection protocols were applied during both patients’ examination and radiographic procedures.

### Sample

The study group consisted of 21 HR patients with an age range of 4 to 16 years. Data on age of diagnosis and medical treatment were collected from medical records of the diseased individuals.

The control group consisted of the same number of age and sex matched healthy children, the panoramic radiographs of the control group were obtained from file records of the orthodontic department at the dental teaching center of Jordan University of Science and Technology which were made for diagnostic purposes.

### Assessment of Dental Maturity

Dental age was assessed from panoramic radiographs using the method of Demirjian et al.^12^ The radiographs were examined under ideal conditions by the same investigator (HA) who was calibrated through daily exercise on random panoramic radiographs using Demirjian’s protocol prior to scoring the study radiographs. Intraexaminer error was assessed by re-examining 10 radiographs on two separate occasions, the difference in dental age was not significant using t-test.

### Statistical Analysis

Statistical analysis was carried out using SPSS-V. 12 program (statistical package for social sciences-Chicago, ILL). Descriptive statistics including mean, standard deviation (SD), and difference between means were calculated for each variable. The difference between chronological and dental age between diseased and healthy individuals was tested using student’s paired t-test. One way t-test was used for the difference between dental and chronological age within the groups. The correlation between amount of dental development delay and age of commencement of treatment was assessed using Pearson correlation. A statistically significant finding was considered present when P-values were below 0.05.

## RESULTS

### Sample

The study population consisted of 21 patients (7 males and 14 females) with HR and 21 healthy controls matched for age and sex. Examining medical records of HR patients revealed that more than half of the sample were diagnosed between age 2 and 12 years, and that all patients started treatment upon diagnosis with oral phosphate supplements at a dose of 50 mg/kg/day, and bioactivated vitamin D at a dose of 40 ng/kg/day.

Table 1 shows the distribution of the dental and chronologic age of males and females in the study population indicating that the difference in dental and chronological age for males and females was almost the same, but the small sample size precluded tests of significance.

### Dental Maturity

Of the 21 HR patients, 14 demonstrated a delay in the development of their dentition ranging from 0.2 to 2.5 years with an average of 0.371 ± 0.72 years delay which was found to be significant using one way t-test (p = 0.028). In contrast, the difference between the chronologic and dental age of the control group was not significant (p = 0.688). The paired t-test showed a statistically significant difference between diseased and controls for their dental age and for the difference between dental and chronologic age (Table 2).

**Fig. 1:**
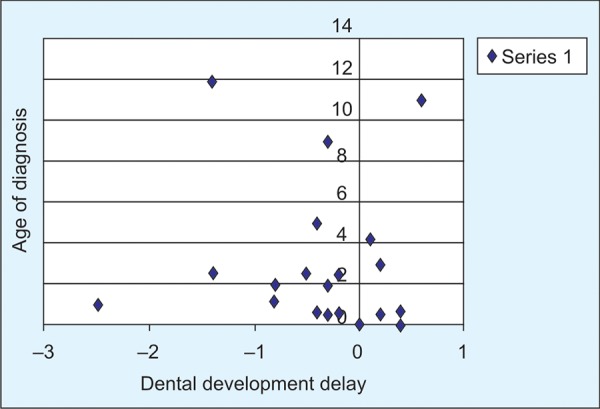
Age at commencement of treatment of HR and the amount of dental delay among the study population

Figure 1 shows the relationship between age of diagnosis of children with HR and dental development. It can be noticed that although some children were diagnosed early, dental delay was still evident. Correlation with age at diagnoses was performed using pearson correlation test which showed no significant association between the amount of delay in dental development and age at commencement of treatment (expressed as percent of patient’s lifetime time without treatment). Assessing the relationship between the amount of delay and the chronologic age of the HR patients demonstrated that as the age of the HR patients increased, the amount of dental development delay also increased (p = 0.026).

## DISCUSSION

The present study showed that patients with HR have statistically significant delay in dental development compare to matched healthy children, a few studies, in the literature, have been conducted on dental development of patients with HR with equivocal results. An old report showed that dental eruption and calcification were normal in a sample of nine patients.^13^ Other case reports in the literature showed altered eruption rate suggesting delay in dental development.^14,15^

The only controlled study was conducted by Seow et al, 1995 who found no statistically significant difference in dental development between diseased and control subjects.^10^ The results of the present study contradict those of the above mentioned study; although the same methodology was used, the age range, mean age and sex distribution were also similar. The reason for this could be due to the fact that most subjects in Seow et al study were diagnosed before the age of two years. In the present study, more than half of the patients were diagnosed at or after age 2 years, which may leave a deficit that will not be corrected later after commencement of treatment. Correlation with age of commencement of treatment in the present report showed no positive effect of treatment on dental development. Many studies on bone growth in these patients found that the growth retardation can be improved but not corrected by obtaining adequate levels of circulating Phosphate.^16^ This persistent deficit in bone growth in these patients was explained by the presence of a primary abnormality in bone responsible for the impaired mineralization in addition to the principle mechanism of abnormal Phosphate homeostasis,^17^ a similar effect may be seen in teeth since dental development may be impaired primarily by hypophosphatemia, or by the secondary effect of rickets on dentine and bone.^10^

**Table 1:** **Table 1:** Distribution of the study population according to age and sex

		*Dental Age* *(mean)*		*SD*		*Range*		*Chronologic age*		*SD* *(mean)*		*Range*	
**HR**													
Male (n = 7)		11.97		1.18		10.3-13.5		12.37		1.92		10.5-	
16.00													
Female (n = 14)		9.15		3.75		4.1-14.60		9.50		3.95		4.4-16.0	
**Controls**													
Male (n = 7)		12.35		2.04		10.7-16.10		12.37		1.91		10.6-	
15.80													
Female (n = 14)		9.62		3.83		4.3-15.9		9.57		3.97		4.0-16.0	

**SD:** Standard deviation

**Table 2:** **Table 2:** Mean dental and chronologic age for HR and healthy controls

*HR Patients*		*Healthy Controls*		*SD*		*t-test*		*Paired t-test* *P-value*			
**Dental Age**		10.09		10.53		0.87		– 2.33		0.030*	
**Chronologic Age**		10.46		10.51		0.20		– 1.07		0.298	
**DA-CA**		0.371		0.023		0.83		– 2.19		0.040*	
**p-value for one way** **t-test within group**		0.028*		0.688							

SD: standard deviation

*Significant

The general limitations of the study included small sample size covering a wide age range, and the need to pool the sexes together, however, small sample sizes are unavoidable, since this disease is rare. Another limitation is the cross sectional nature of the study.

Longitudinal studies can help determine weather the deficit noted is true and persistent or that treatment can result in correction of the deficit. The population of the present study are under follow-up and their dental development will be reported longitudinally in a separate paper.

## CONCLUSION

Given the limitations of the present study, the results suggest that HR adversely affects dental development and that delay in commencement of medical treatment may lead to a permanent deficit in dental development. Further longitudinal studies are needed to validate the results.
